# Development and assessment of feasibility of a community-based peer support intervention to mitigate social isolation and stigma of adolescent motherhood in Harare, Zimbabwe

**DOI:** 10.1186/s40814-021-00832-0

**Published:** 2021-05-17

**Authors:** Chiwoneso B. Tinago, Edward A. Frongillo, Andrea M. Warren, Vivian Chitiyo, Ashley K. Cifarelli, Shannon Fyalkowski, Victoria Pauline

**Affiliations:** 1 Department of Health, West Chester University of Pennsylvania, 855 S. New Street, West Chester, PA 19383 USA; 2 Department of Health Promotion, Education, and Behavior, Arnold School of Public Health, University of South Carolina, 915 Greene Street, Room 529, Columbia, SC 29208 USA; 3 The Organization for Public Health Interventions and Development (OPHID), 20 Cork Road, Belgravia, Harare, Zimbabwe

**Keywords:** Adolescent mothers, Zimbabwe, Mental health, Adolescent health, Maternal health, WhatsApp Messenger

## Abstract

**Background:**

Adolescent mothers in Zimbabwe often experience stigma, isolation, and lack coping skills and resources to successfully navigate motherhood. Social isolation and stigma are linked to poor mental health outcomes. No interventions currently address mental health of adolescent mothers in Zimbabwe. Peer support groups in other contexts have been effective at increasing social connectedness, self-esteem, and self-efficacy, providing coping mechanisms to manage stigma experiences, in addition to empowering and improving mental health of adolescents and adolescent mothers. To develop a community-based peer support intervention, we aimed to understand the unique needs of adolescent mothers, how peer support groups could address those needs, and the feasibility of implementing the intervention.

**Methods:**

Focus group discussions were conducted with 86 adolescent mothers aged 14–18 years, 24 community health workers, and 25 key community stakeholders in a low-income high-density community in Harare. Data were analyzed thematically using NVivo 12 software.

**Results:**

Participants described adolescent mother experiences with stigma and social isolation, in addition to challenges including gossip, lack of employment and educational opportunities, and gaps in services and programming. Peer support groups for adolescent mothers were welcomed to improve mental health, social support, knowledge sharing, and skills building. Participants identified varying preferred frequency and duration of group sessions addressing topics including income generation, mental health, and gossip, facilitated by community health workers at health and community centers. The use of WhatsApp Messenger to support intervention efforts was welcomed as an affordable and user-friendly platform to share information. Implementation (i.e., training, supervision, frequency, location, and co-facilitation) was feasible.

**Conclusions:**

Adolescent mothers, community health workers and key community stakeholders welcomed the peer support groups as a feasible way to address the mothers’ needs.

## Key messages regarding feasibility

- Adolescent mothers in Zimbabwe experience challenges that stem from social isolation and stigma and negatively influence health. Adolescent mothers and community members endorse a peer support intervention for improving the mental health of adolescent mothers.
- This study solicited perspectives from adolescent mothers, health workers, and key community members to inform the development of a peer support intervention that was subsequently piloted by the study team.
- Implementation of the intervention regarding training and supervision of facilitators and frequency, location, and co-facilitation of the peer support groups was feasible.

## Background

Almost a quarter of adolescent girls aged 15–19 years in Zimbabwe have begun childbearing [1]. These pregnancies are often unintended, due to early marriage, sexual abuse, or risky sexual behavior [2]. There is stigma related to adolescent pregnancy and motherhood in Zimbabwe with adolescent mothers often feeling isolated due to lack of social support with a loss of social networks and educational opportunities. Adolescent pregnancy in Zimbabwe leads to dropping out of school (19.4%), being abandoned by friends (4.9%) and the person responsible for the pregnancy (9.1%), and/or being forced to leave home (57.7%) and marry at a young age (24.5%) [1, 2]. In Zimbabwe, adolescent mothers dismissed from school have difficulty returning to the educational system. The stigma of adolescent pregnancy and motherhood is well documented in both developing and developed countries with stigma experienced from both within the family, from peers, institutions such as schools and health facilities and by the community at large [3–6].

Social isolation and stigma are linked to poorer mental health outcomes such as increased depressive and anxiety symptoms, suicidal ideation, and suicide attempts [3, 4, 7]. Adolescent mothers may also lack coping skills and resources to successfully navigate motherhood [3, 5, 6]. Unless addressed, these circumstances may have negative consequences for the mental health of the adolescent mother and downstream consequences for her child.

Zimbabwe has a prevalence above 20% for common mental disorders. Depression and anxiety are reported in over 25% of those attending primary health and maternal services and in up to 30% of females in the community [8–10]. The peak onset of depression occurs between adolescence and young adulthood [11, 12]. Early-onset depression has a 60–70% risk of continuing into adulthood [13]. Adolescents suffering from depression are at greater risk for self-harm, conduct disorders, delinquency, and high-risk behaviors such as substance use and early sexual debut [14–16]. The national prevalence of depression among Zimbabwean adolescents is unknown [17]. The prevalence of suicide ideation among school-going adolescents in Zimbabwe is 21.6% [18].

Zimbabwe faces human-resource shortages for mental neurological and substance use disorders and care with only 12 psychiatrists and 16 psychologists [19]. The number of programs and interventions that address adolescent mental health in Zimbabwe is limited, and these target children and adolescents with HIV [20]. No interventions target adolescent mothers. Programs and interventions are needed to prevent early pregnancies, meet the needs of adolescent mothers, and improve their health.

Globally, peer support group interventions have been effective at increasing social connectedness, self-esteem, and self-efficacy, providing coping mechanisms to manage stigma experiences, in addition to empowering and improving adolescent and adolescent mother mental health and emotional well-being [21–27]. Peer support groups provide a platform for knowledge and experience sharing to provide emotional, social and practical support [21–27]. Peer support groups implemented virtually via mobile messaging apps or online platforms have also documented increased social connectedness, stigma coping mechanisms, and positive mental health and social support outcomes for adolescents and adolescent mothers [27, 28]. In a peer support intervention based on mobile phones where adolescent mothers in Canada received support from a trained peer mentor by voice calling or text messaging during their last trimester of pregnancy and 12 weeks postpartum, participants in the intervention group demonstrated lower mean depression scores at 12 weeks postpartum compared to participants in the control group [28].

Participatory program development leads to better uptake and alignment with local institutional capacities and resources [29]. Interventions such as the Paying Attention to Self-intervention in Australia that involved adolescents in the development and implementation of these groups, in addition to involving other key social networks such as family members, were also successful at improving adolescent mental health and emotional well-being [23].

Because most interventions for adolescent mothers have been conducted in high-income countries, little is known about whether such interventions will similarly improve mental health in low- and middle-income countries. A limitation of completed programs in high-income countries has been not having feedback from participants who leave the interventions [29].

To develop a community-based peer support intervention to mitigate social isolation and stigma of adolescent motherhood and improve mental health of adolescent mothers in Harare, Zimbabwe, we conducted participatory formative research with adolescent mothers, community health workers and community members, aiming to understand 1) the unique needs of adolescent mothers, 2) how, from participant perspectives, peer support groups could address those needs, and 3) the feasibility of implementing the developed intervention.

## Methodology

### Setting

Zimbabwe is in south-east Africa and has a population of 12,973,808 (51.9% females) [30]. The study was conducted in a high-density, low-income, peri-urban community in Harare. English is the official language of Zimbabwe, and Shona is the main local language in Harare.

### Design

A qualitative descriptive research design with an emic orientation was used with focus group discussions (FGDs) to learn about participants’ perspectives concerning specific topics that could be addressed through a peer support group for adolescent mothers (14–18 years) and the preferred structure and methods for conducting the groups [31, 32]. The reporting of qualitative research follows the Consolidated Criteria for Reporting Qualitative Research [33].

### Sample

The sample was 86 adolescent mothers (pregnant and/or have a child or children) aged 14–18 years living in the study community (10 FGDs), 24 community health workers (CHWs) who worked in the study community (3 FGDs), and 25 key community stakeholders in the study community consisting of teachers, family members of adolescent mothers, health officials, and religious leaders (2 FGDs). Thematic saturation was expected based on previous qualitative studies in similar populations with a sample size of at least 24 participants [34, 35].

### Recruitment

The purposive sample was recruited in locations such as community centers, pre- and postnatal clinics, and churches. Participants were recruited by CHWs and through fliers and snowball sampling. Participants were screened for eligibility to participate in the FGDs, with the criteria being (a) adolescent mothers (pregnant and/or have a child or children) aged 14–18 years living in the study community, (b) CHWs who worked in the study community, and (c) key community stakeholders in the study community consisting of teachers, family members of adolescent mothers, healthcare workers, health officials, and religious leaders. Participants were provided with a consent form that disclosed the credentials and positions of the study team and explained what the data would be used for and the intended outcome of the study. Participants provided written consent; additional parental or guardian consent was provided for participants aged 14–17 years.

### Data collection

FGDs with 135 adolescent mothers, CHWs, and key community stakeholders were conducted between October–December 2018 to determine the peer support group structure, topics for discussion, meeting times, and frequency. A FGD guide (with open-ended questions and subsequent probes), socio-demographic survey, and consent and assent forms were developed in English and professionally translated to Shona. The study team identified WhatsApp as a feasible platform through which to deliver intervention components in a previous study with adolescent females in the same study community, so a portion of the FGDs focused on local usage of WhatsApp to confirm its feasibility. The FGD guide was pretested in October 2018 and revised to ensure comprehension of questions. FGD questions included 1) when you think about the health of adolescent mothers, what comes to mind? 2) what can be done to improve the health of adolescent mothers in your community? 3) if there was a peer support group for adolescent mothers, what would this group look like? Further questions asked who should be involved; what information and resources would be helpful; who should be targeted; who should deliver information and resources; by whom, where, and when should they be delivered; length and frequency of sessions; and what the groups should be called. Feasibility, i.e., how well technical complexity and capacity matched, was assessed through the FGD with CHWs by asking about sufficiency of purpose of training and supervisory support provided and challenges they encountered regarding the frequency, location, or co-facilitation of the groups [36].

FGDs were conducted at the community clinic and community hall. After providing consent or assent, participants completed a brief interviewer-administered socio-demographic survey immediately prior to the FGD. FGDs were 60 min each with 6–12 participants in each group. Only the study team and participants were present during the FGDs. Field notes were taken during and immediately following the FGDs. FGD data were collected by the project coordinator, principal investigator, and a trained research assistant who are female and fluent in Shona and English. Study team members have undergraduate and/or post-graduate training in qualitative methods and have experience conducting qualitative research. FGDs were audio-recorded, professionally transcribed verbatim, and translated to English.

### Data analysis

Socio-demographic data were analyzed with Stata 13. Transcripts were analyzed thematically between November 2018 and May 2019 using NVivo 12 software [37]. The coding process was both inductive and deductive [37]. The principal investigator and two trained research assistants developed an initial codebook with deductive codes based on questions asked in the FGD guide related to the format of the peer support groups. The data analysts then coded 4 transcripts with the initial codebook and open coded these transcripts with any new codes that emerged. Data analysis meetings were conducted to discuss and revise the preliminary code book with the inductive and deductive codes. The data analysts then recoded the initial 4 transcripts and an additional 7 transcripts with the revised code book. Weekly data analysis meetings continued, and the codebook was revised based on the additional coding until saturation was reached. Interviewer and data analyst triangulation was used to prevent threats to validity inherent in using a single interviewer and data analyst [38]. Participant quotes are identified by FGD number and participant type, and abbreviations are used to identify adolescent mother (AM), community health worker (CHW), and key community stakeholder (KCS).

## Results

### Sample characteristics

#### Adolescent mothers (*n*=86)

The mean age was 17.6 years (range 15–18), and most were of Shona ethnicity (96.5%) (Table 1). Most were in married monogamous relationships (54.7%) and received high school education between Form 1 and 4 (82.6%); 41.2% were Pentecostal, 50% were dependent on their partner/family for income, and 88.4% had a previous pregnancy. Most had other children (74.4%), and the average number of other children was 1.1 (range 0–4), and 40.7% gave birth to their recent child in the hospital. Fewer were pregnant at the time of screening (17.4%), and most families knew of the pregnancy (86.7%). The mean months pregnant was 5.8, and 53.3% had attended an antenatal care visit with the first visit occurring on average at 4.6 months. Mean number of visits was 2.4 (range 1–4) and 50% attended a monthly visit. Most had a cell phone (73.3%) and used WhatsApp (58.7%).

**Table 1:** Socio-demographic characteristics of focus group study participants (*N*=135)

Characteristic	Adolescent mothers (***n***=86)	Community health workers (***n***=24)	Key community stakeholders (***n***=25)
	***n*** (%)	***n*** (%)	***n*** (%)
**Gender**			
Female Male	86 (100.0) 0 (0)	23 (95.8) 1 (4.2)	19 (76.0) 6 (24.0)
**Age**			
Mean age (years) Age range (years) Missing	17.6 15–18 0	60.3 37–77 1 (4.2)	45.7 18–61 2 (8.0)
**Ethnicity**			
Shona Other	83 (96.5) 3 (3.5)	24 (100.0) 0	23 (92.0) 2 (8.0)
**Marital status**			
Divorced or separated Married—monogamous Married—polygamous Never married Widowed	13 (15.1) 47 (54.7) 4 (4.7) 22 (25.6) 0	0 10 (41.7) 0 0 14 (58.3)	5 (20.0) 7 (28.0) 4 (16.0) 3 (12.0) 6 (24.0)
**Highest education level**			
Primary (grades 1–7) High school (forms 1–4) Some College or University Bachelor’s Other None Missing	14 (16.3) 71 (82.6) 0 0 0 0 1 (1.2)	1 (4.2) 4 (16.7) 3 (12.5) 1 (4.2) 15 (62.5) 0 0	3 (12.0) 20 (80.0) 0 0 1 (4.0) 1 (4.0) 0
**Religious affiliation**			
Apostolic Christian Traditional Other Missing	17 (19.8) 65 (75.6) 0 3 (3.5) 1(1.2)	2 (8.3) 20 (83.3) 1 (4.2) 1 (4.2) 0	4 (16.0) 21 (84.1) 0 0 0
**Main source of income**			
Self-employed Formerly employed Dependent None Other Missing	7 (8.1) 3 (3.5) 43 (50.0) 22 (25.6) 2 (2.3) 1 (1.2)	**-** **-** **-** **-** **-** **-**	**-** **-** **-** **-** **-** **-**
**Current employment**			
Yes No	- -	24 (100.0) 0	6 (24.0) 19 (76.0)
**Prior pregnancy**			
Yes No Missing	76 (88.4) 9 (10.5) 1 (1.2)	- - -	- - -
**Number of prior pregnancies**			
1 2 3 4 None Missing	61 (70.9) 8 (9.3) 1 (1.2) 1 (1.2) 5 (5.8) 2 (2.3)	- - - - - -	- - - - - -
**Children**			
Yes No Missing	64 (74.4) 12 (14.0) 0	- - -	- - -
**Number of children**			
Average Range	1.1 0–4	- -	- -
**Place of last birth**			
Home Clinic Hospital	1 (1.2) 28 (32.6) 35 (40.7)	- - -	- - -
**Currently pregnant**			
Yes No Missing	15 (17.4) 68 (79.0) 3 (3.5)	- - -	- - -
**Family know you’re pregnant**			
Yes No Missing	13 (86.7) 1 (6.7) 1 (6.7)	- - -	- - -
**Months currently pregnant**			
Average Range Missing	5.8 3–9 2 (2.3)	- - -	- - -
**Attended ANC**			
Yes No Missing	8(53.3) 6(40.0) 1(6.7)	- - -	- - -
**Months pregnant first ANC**			
Average Range Missing	4.6 3–7 3 (3.5)	- - -	- - -
**Number of ANC visits**			
Average Range	2.4 1–4	- -	- -
**Frequency of ANC**			
Every month Other Missing	4 (50.0) 2 (25.0) 2 (25.0)	- - -	- - -
**Cellphone**			
Yes No Missing	63 (73.3) 19 (22.1) 4 (4.7)	- - -	- - -
**WhatsApp**			
Yes No	37 (58.7) 26 (41.3)	- -	- -

#### Community health workers (*n*=24)

Mean age was 60.3 years (range 37–77) and 37.5% were Protestant. Most were widowed (58.3%), females (95.8%), of Shona ethnicity (100.0%) that resided in the study community (83.3%). Most had other education (62.5%), followed by high school form 1–4 education (16.7%). They had an average of 27.9 years of experience as a community health worker (range 15–32) who worked with young women (96.0%).

#### Key community stakeholders (*n*=25)

The mean age was 45.7 years (range 18–61), most were women (76.0%), of Shona ethnicity (92.0%), and resided in the study community (92.0%); 28.0% were Pentecostal, and 28.0% were monogamously married. Most had a high school form 1–4 education (80.0%) and were not currently employed (76.0%); 44.0% worked with young women, and 60.0% had a female child of their own.

### The challenges of adolescent motherhood

Most participants described adolescent motherhood in terms of the challenges of adolescent motherhood that stemmed from experiences with social isolation and stigma and led to feelings of social disconnectedness, disempowerment, lack of control, abuse, risky behaviors, and poor health. Participants described challenges that centered on poverty and lack of employment opportunities with negative influences on health. An adolescent mother stated, “Most of the time we won’t have any money” (AM, Group 5). The lack of money was often a result of most adolescent mothers not finishing their education due to expulsion from school for their adolescent pregnancy or lack of family financial support to continue their education which then limited their employment prospects. A key community stakeholder explained:

Another issue is that these adolescents have not completed their education. So their brains, if they had gone maybe up to form 4, O Level (high school grade 11) they would have been mature and by going to school we meet teachers and learn different things. So being uneducated is also a problem again because they won’t really know what the future holds…If they could be taken back to school it would be nice. (KCS, Group 2)

Participants described financial worries leading to risky behaviors and poor health with a community health worker stating, “I think the main issue is on poverty, this will lead them to do everything and anything. They see what others would be having and start saying how can I get that. They end up getting into difficult positions which she was not supposed to due to poverty” (CHW, Group 1). These difficult positions included substance abuse and risky behaviors for financial compensation. A community health worker described:

Health with regards to adolescent mothers what we see is that they don’t do good to themselves, if they see that they have been given a child, let’s say they are a single mother, they no longer take care of themselves. They start smoking and drinking beer, they may actually go to beer halls thinking that they may get something, the beer hall is not good for health. They may find a lot of things that will trouble them, smoking will cause her to be sick and having so many boyfriends will cause her to be infected (with HIV). (CHW, Group1)

An adolescent mother added, “Our health can be affected through worrying on how we can take care of our children, money is a problem. Others don’t have anything to do that can give us money so our health can be affected through thinking about getting something to do” (AM, Group 10). Another adolescent mother added, “I think if adolescent mothers can work for themselves their health would be improved” (AM, Group 7) while a key community stakeholder described, “Something should be done for women for them to be able to earn a living and to empower them. We need to be taken to another level, out of the mud that we are currently in” (KCS, Group 2).

Participants also described challenges with adolescent motherhood due to marriages with a participant stating, “If you have your own money that would be better, a lot are not getting into marriages by choice, it is driven by that idea that maybe my life will be changed but you end up seeing that your life is actually getting worse” (AM, Group 4). The participant was referring to the cultural practice of brides price or “lobola” paid by the groom to the bride’s family. Another adolescent mother added:

It’s so difficult to be a mother while you are still young because what you face in the marriage, its different from someone who is married at 25 years, that person will be mature and knowledgeable. You might come across a situation let’s say the husband does not go to work, it becomes difficult for you as you have to provide for yourself. So it is so difficult being a mother while you are still young…life is just difficult if you are the mother of the house. (AM, Group 8)

Experiences with stigma and social isolation from family, peers, partners, and community members and their influence on health were also provided by participants as they described being chased away from home by relatives, being left by the person who impregnated them, and abuse as negatively affecting adolescent mother’s mental health. An adolescent mother stated, “To be impregnated and someone leaves you is such a painful thing so you won’t have mental stability” (AM, Group 7). Another adolescent mother described, “Being abused by a husband can cause you to have bad mental health, you will always think about what your tomorrow will be like if I spend of my days being abused and beaten, it causes you to be always thinking” (AM, Group 1). A community health worker added:

The other thing that causes them not to run to relatives is because they are too harsh with them. She would have gone to tell them about her situation, and she is beaten up. She is chased away from the house and is told to go back to the boyfriend who may not be stable. The adolescent mother becomes stress that is when you see her becoming suicidal or hurting herself because she will be overwhelmed with the problem, the relatives would have chased her away. That is why she looks for refuge outside to us as community health workers because she knows that we will give her advice on way forward. (CHW, Group 1)

### Gaps in adolescent mother services and programming

Most participants identified gaps in services and programming for adolescent mothers in their community. An adolescent mother stated, “There is need even for building of play centers where people can meet and socialize relieving their stress as women” (AM, Group 1) and another added:

If they could get some projects that they can do as this reduces their stress because if you are at work you are occupied with what you have to do than spending the whole day seated, that’s when you start having stress…So if you have something to occupy you that would be better. (AM, Group 1)

Peer support groups for adolescent mothers were welcomed to meet needs of adolescent mothers and engage them in activities with peers for social support, problem-solving, knowledge sharing, and skills building. An adolescent mother stated, “I think us as young people should get the opportunity to get into groups educating each other, people giving each other advice. I think that will help us as adolescent mothers on how we can live” (AM, Group 8). A key community stakeholder explained:

I think support groups is something they would really like because they know that they will get something out of it. You don’t get something while you are just seated at home, you can only get this when you are in groups. Forming support groups is a good way we can mobilize these children. (KCS, Group 1)

### Peer support group structure

#### Facilitators

Participants described that peer support groups should be delivered by trusted and informed people such as nurses and adults with health knowledge. An adolescent mother stated, “These can be delivered by someone whom we trust that they can get back to us with an answer, we would have put all our trust in that person” (AM, Group 6). Another adolescent mother added, “The nurse from the clinic should be able to help us” (AM, Group 6), while another explained, “I think we would need adults who know about health” (AM, Group 3). Key community stakeholders reiterated the need for older adults to facilitate the groups with a stakeholder stating, “Older women and even men can be part of the group...if there are adults in there, there will be some order” (KCS, Group 1).

#### Location

Most participants preferred that the peer support groups be conducted at the local clinic, “They should be delivered here at the clinic” (AM, Group 5). Participants described clinics, along with community hall, schools, and churches, as central and trusted settings.

#### Frequency

The preferred frequency of peer support group sessions varied from meeting every day to once a month. An adolescent mother stated, “I think it should be done on a monthly basis” (AM, Group 3), while another explained, “If we are brought together as young people and we meet at least twice per week we can be able to learn, get to know each other and share information” (AM, Group 5). Another adolescent mother described, “I think every week, let’s say we meet once a week, by the end of the month we would know that we have four days that we meet educating and encouraging each other, doing our things as a unit” (AM, Group 8).

#### Duration

Participants described that peer support groups should meet about two hours at a time, over varying periods of time. Responding to how long peer support groups should be offered, an adolescent mother replied, “Two hours” (AM, Group 3). Participants wanted the peer support groups to be offered continuously with an adolescent mother describing, “As long as forever, the whole life because more and more adolescent mothers are being added every day” (AM, Group 1). Another adolescent mother added, “We would want them to be around for a long time so that they can assist even other adolescent mothers who will come after us” (AM, Group 10). Shorter term group meetings were also described with adolescent mothers suggesting that groups, “…can go to up to a year” (AM, Group 2) and “6 months” (AM, Group 4).

#### Topics

Participants described topics that included income generation, life skills, sexual health, mental health, gossip, hygiene, abuse, and breastfeeding. An adolescent mother described, “We would like to be helped with money so that we can be able to carry out these projects for a living” (AM, Group 10). Empowerment by means of income generation and collaboration was described a way to increase their locus of control and support themselves. A community health worker added, “I think if projects could be done for them so that they may have time to come together doing something together and their minds can be stabilized as they work for themselves, I think that could work. Because some of them do not know how to work for themselves” (CHW, Group 1). Participants described how risky behaviors could be prevented with additional knowledge on substance abuse and risky sexual behavior. An adolescent mother stated, “Maybe on drug abstinence because many adolescent mothers are now abusing drugs” (AM, Group 7). A key community stakeholder added, “I think on sexual and reproductive health, which is one topic that is very important for these young people” (KCS, Group 1).

Adolescent mothers described rumors spread about them and personal information shared without their consent through gossip as interfering with their communication with peers and community members. Gossip was also characterized as a contributor to the stigma of being an adolescent mother along with experiences of social isolation and depressive symptoms. An adolescent mother described:

If you hear something, let’s say your brother’s daughter has come to tell you her problem, you should not say as soon as she tells you, you are taking that to the next person. Then you hear that from so and so to say we heard this about you, that way we won’t be in good books with each other. So what we are asking is if you could go out there and educate them that they should not say if we come with our problems to them they go out spreading the word, selling it everywhere. (AM, Group 9)

Another adolescent mother explained:

You are walking in the road and you approach a group…you fear to say “should I pass through” or I should change the route because they will remain behind talking about me. They may even talk loudly so that you can hear and laugh at you. This can actually cause pain in you to the extent that you regret why you used that route. (AM, Group 1)

Adolescent mothers described gossip as leading to mistrust with an adolescent mother stating, “As for me…to tell my relative or to share my problems with them, they will share my secret. They are a gossiper” (AM, Group 1). Another adolescent mother stated that the reason she did not share information with adults in her life was because “some of them don’t keep secrets” (AM, Group 6).

#### Name

Most participants named the peer support groups “Young Women of Today” (*Madzimai eChidiki Anhasi*) to emphasize their identity as young women first, then mothers who face current issues.

### WhatsApp Messenger use

#### Likes of WhatsApp

Use of WhatsApp Messenger to support intervention efforts was welcomed by most participants. Features that participants liked about WhatsApp were its ease of communication and affordability; how it allows information to travel fast; and the ability connect with others to share ideas, problem solve, get help, and participate in group discussions, provide amusement, and learn. An adolescent mother explained, “If someone has encountered a problem at home we could use the WhatsApp groups to share ideas, people can help you here and there without waiting to meet in person, people can assist you fast” (AM, Group 1). Another adolescent mother added, “WhatsApp is good because if you don’t have enough money to phone you can talk through WhatsApp because if you juice up a dollar you can go for a week talking” (AM, Group 2).

#### Dislikes of WhatsApp

Features that most participants disliked about WhatsApp were chain messages, inappropriate messages, network challenges, identification of phone number, overuse, hacking of account, and its role in spreading misinformation. An adolescent mother stated, “Someone might actually be saying bad things in those groups which does not go in line with the purpose of the group” (AM, Group 3), while another added, “Bad influence, gossip, sharing bad videos” (AM, Group 4). A community health worker explained, “I don’t like chain messages and some messages you just delete before reading” (CHW, Group 1).

#### Frequency of WhatsApp use

Participant dislikes about WhatsApp did not limit their use of WhatsApp. WhatsApp Messenger was used frequently among those who had the messaging app with adolescent mothers stating they used WhatsApp “The whole day” (AM, Group 1) and “I think I can use it for almost 14 hours of my day” (AM, Group 2). The frequent use of WhatsApp was a way to increase their social connectedness and as a resource for information and entertainment.

#### Reasons for WhatsApp use

Most participants described that they used WhatsApp for communicating with family and friends, church groups, and for learning and current affairs/news. An adolescent mother stated, “Most of the time I will be talking to people in our church group” (AM, Group 8) and another added, “I have WhatsApp groups from church where we are updated on what is happening, family groups and other relatives. It is easy to communicate with than phoning” (AM, Group 1). An adolescent mother stated, “I have a number of groups that I am part of, for novels and cooking. So if I see that I have received quite a number of messages on the novels, I switch off data and start going through the novels. I can also copy a recipe that I want from the group” (AM, Group 6). Another adolescent mother explained:

We can use it telling each other good information. Like what we were saying if there is one of us who has got a challenge, she can share it on the group. As adolescent mothers we should encourage each other, not laugh at her. That is what we would like to use WhatsApp for, to encourage each other as young people. (AM, Group 5)

### Feasibility of implementation

Facilitators reported that the detailed training and supervisory support provided (both in-person and via WhatsApp) were sufficient for purposes, and reported no significant challenges related to the frequency, location, or co-facilitation of the groups. All expressed a strong desire for the intervention to continue.

## Discussion

Participants described the challenges of adolescent motherhood that included poverty and lack of employment opportunities, early marriages, being chased away from home by relatives, being left by the person who impregnated them, and abuse. Participants also identified gaps in services and programming for adolescent mothers in their community.

Peer support groups for adolescent mothers were welcomed to meet social support, health, coping, empowerment, skills building, and educational needs of adolescent mothers. Participants identified potential topics that included income generation, gossip, mental health, sexual and reproductive health, abuse, and hygiene. Income generation was the most stated topic and was related to participants’ inability to continue their education and thus find employment to support themselves and their families. Similarly, Carbone and colleagues (2019) used FGDs with adolescent mothers aged 15–19 in Malawi to inform the development of a program to educate on the prevention of mother-to-child transmission of HIV [39]. Adolescent mothers in their study requested education on subjects not directly related to transmission, such as poverty, food insecurity, economic disempowerment, and financial dependence, which were often cited as barriers to care. These findings corroborate ours about the quest for economic empowerment together with health-related interventions among adolescent mothers in low income settings.

Rumors spread about adolescent mothers and secrets shared without their consent through gossip from peers and community members led to adolescent mothers feeling isolated, stigmatized, and mistrustful. Gossip also led to adolescent mothers experiencing depressive symptoms due to the pain caused by these rumors and violation of trust. Adolescent mothers in Zambia also found returning to school difficult due to expulsion, stigmatization, gossip, and isolation from peers and community members; discrimination and stigmatization of adolescent motherhood from the community led many adolescents into early marriages, with some engaging in risky behaviors such as substance abuse to cope with stigmatization [40].

Participants emphasized that peer support groups should be delivered by trusted and informed people such as nurses and adults with health knowledge in trusted settings such as local health and community centers. Community health workers and community stakeholders emphasized having an adult facilitate the peer support groups. The preferred frequency of peer support group sessions varied from meeting every day to once a month. Adolescent mothers named the peer support groups “Young Women of Today” to emphasize their identity as young women first, then mothers who face current issues.

Use of the WhatsApp messaging app to support intervention efforts was welcomed as an affordable, convenient, and user-friendly platform to share information if the messaging platform was private and not used to spread gossip, misinformation, and chain messages. Hay and colleagues (2020) reported similar barriers and facilitators to WhatsApp use among mentor mothers with HIV in the UK with additional barriers related to limited and costly data storage [40]. Henry and colleagues (2016) implemented a mobile learning intervention to enhance the supervision of community health workers with WhatsApp mobile messaging in 2 low-resource settings in Kenya and found that with minimal training, community health workers and their supervisors were able to conduct virtual one-to-one, group, and peer-to-peer supervision via WhatsApp [41]. More evidence is needed to explore the effectiveness of WhatsApp as an intervention tool.

A limitation of our study is that the perspectives reported in the focus groups may incompletely convey the needs of individual young women. Strengths of our study include the large representative sample of adolescent mothers, key community stakeholders, and community health workers who provided community perspectives on peer support groups for adolescent mothers and the use of multiple data analysts to offset threats to validity.

## Conclusion

Participants welcomed the idea of a community-based peer support intervention to mitigate social isolation and stigma of adolescent mothers by way of integrated technology, peer and community support, empowerment, skills building, and knowledge sharing. Implementation of the intervention in terms of frequency, location, and co-facilitation of the peer-support groups was feasible and training and supervision of facilitators was sufficient. Facilitated peer support groups can strengthen social networks and mitigate the adverse effects of social isolation and stigma among adolescent mothers. Integral to these efforts is a focus on participatory methods that empower adolescent mothers to inform the development of programs and interventions that address their health and social needs.

## Data Availability

The datasets used and/or analyzed during the current study are available from the first author on reasonable request.

## Abbreviations

CHW: Community health worker
FDG: Focus group discussion
AM: Adolescent mother
KCS: Key community stakeholder
